# Ventilated Patients With COVID-19 Show Airflow Obstruction

**DOI:** 10.1177/08850666211000601

**Published:** 2021-03-11

**Authors:** Vikas S. Koppurapu, Maksym Puliaiev, Kevin C. Doerschug, Gregory A. Schmidt

**Affiliations:** 1 Division of Pulmonary and Critical Care Medicine, Department of Medicine, 21782 University of Iowa Hospitals and Clinics, Iowa City, IA, USA

**Keywords:** COVID-19, mechanical ventilation, atypical, respiratory mechanics, obstruction, airway resistance

## Abstract

**Objective:**

Many patients with coronavirus disease 2019 (COVID-19) need mechanical ventilation secondary to acute respiratory distress syndrome. Information on the respiratory system mechanical characteristics of this disease is limited. The aim of this study is to describe the respiratory system mechanical properties of ventilated COVID-19 patients.

**Design, Setting, and Patients:**

Patients consecutively admitted to the medical intensive care unit at the University of Iowa Hospitals and Clinics in Iowa City, USA, from April 19 to May 1, 2020, were prospectively studied; final date of follow-up was May 1, 2020.

**Measurements:**

At the time of first patient contact, ventilator information was collected including mode, settings, peak airway pressure, plateau pressure, and total positive end expiratory pressure. Indices of airflow resistance and respiratory system compliance were calculated and analyzed.

**Main Results:**

The mean age of the patients was 58 years. 6 out of 12 (50%) patients were female. Of the 21 laboratory-confirmed COVID-19 patients on invasive mechanical ventilation, 9 patients who were actively breathing on the ventilator were excluded. All the patients included were on volume-control mode. Mean [±standard deviation] ventilator indices were: resistive pressure 19 [±4] cmH_2_O, airway resistance 20 [±4] cmH_2_O/L/s, and respiratory system static compliance 39 [±16] ml/cmH_2_O. These values are consistent with abnormally elevated resistance to airflow and reduced respiratory system compliance. Analysis of flow waveform graphics revealed a pattern consistent with airflow obstruction in all patients.

**Conclusions:**

Severe respiratory failure due to COVID-19 is regularly associated with airflow obstruction.

## Introduction

In December 2019, the first patients with pneumonia due to a new species of coronavirus (novel coronavirus, now named SARS-CoV-2) were admitted to hospitals in Wuhan, China.^1^ Since then, SARS-CoV-2 virus has spread across the world, leading to a pandemic of the associated disease, COVID-19. A significant proportion of patients with COVID-19 develop severe respiratory disease and may require admission to the intensive care unit (ICU) and mechanical ventilation.^2–4^

Patients with COVID-19 pneumonia requiring mechanical ventilation typically fulfill the diagnostic criteria for the acute respiratory distress syndrome (ARDS).^5^ Some authors pointed out that a substantial fraction of patients with COVID-19 pneumonia exhibit features quite atypical for ARDS. For example, gas exchange may be impaired out of proportion to mechanical abnormalities (“happy hypoxia”)^6^ and respiratory system compliance is relatively preserved.^7^ Gattinoni and colleagues have hypothesized 2 distinct phenotypes of lung failure in COVID-19: L-type with low elastance and H-type with high elastance.^8^ These peculiar features may affect treatment choices, such as levels of positive end expiratory pressure (PEEP) or strategies for delivering lung-protective ventilation. More fundamentally, these characteristics offer clues to pathophysiology of lung failure in COVID-19 pneumonia and could point to new therapies.

In our experience managing patients with COVID-19, we noticed another unusual feature: airflow obstruction was typically evident. Ventilator graphics display pressure and flow waveforms as a function of time, allowing for prompt recognition of this phenomenon. The typical findings of airflow obstruction, including elevated peak airway pressure, abnormal resistive pressure (peak minus plateau airway pressure, P*resist*), and an abnormal expiratory flow profile (low expiratory flow rate with flow persisting late in the expiratory phase, sometimes with evident end-expiratory flow) were present so often that we hypothesize that airflow obstruction is an integral part of COVID-19-related lung failure.

## Materials and Methods

### Ethics

The project was approved by the Institutional Review Board (IRB). Waiver of informed consent was granted.

### Patient Selection, Setting, and Data Acquisition

Adult subjects with ARDS due to COVID-19 pneumonia ventilated in the medical ICU between April 19 and May 1, 2020 were prospectively identified during usual ICU care. ARDS was defined according to the Berlin criteria. All subjects were passively breathing on volume-control mode at the time of contact. A heat moisture exchange (HME) filter was used in all ventilator circuits. Ventilator settings and patient-ventilator interaction measurements were obtained and documented in the electronic health record (EHR), as were screenshots of ventilator waveforms. The following additional data were collected:

- Patient information including age, sex, virologic testing results including SARS-CoV2 and respiratory viral panel, duration of mechanical ventilation, body mass index (BMI), history of prior lung disease, smoking history and prior pulmonary function test results.
- Prone vs supine position.
- Use of neuromuscular blocking agents (NMB).
- Ventilator information including settings (tidal volume (V*t*), respiratory rate, fraction of inspired oxygen (FiO2), positive end-expiratory pressure (PEEP)), peak pressure (P*peak*), plateau pressure (P*plat*), total PEEP (PEEP*tot*), and ventilator graphics.

Institutional recommendations were consistent with evidence-based care for ARDS, including low tidal volume ventilation, maintenance of plateau pressures less than 30 cm H_2_O, driving pressure of 15 cm H_2_O or less, and conservative fluid therapy. Prone positioning was utilized for severe hypoxemia (e.g. ratio of partial pressure of oxygen on arterial blood gas to fraction of inspired oxygen (PaO_2_/FiO_2_) less than 150, or ratio of oxygen saturation on pulse oximetry to FiO2 (SaO_2_/FiO_2_) less than 180). NMB was initiated for significant patient-ventilator dyssynchrony. PEEP titration and other aspects of patient management were at the discretion of the ICU attending physician.

### Measurements and Definitions

The P*plat* and PEEP*tot* were measured by performing manual inspiratory and expiratory hold maneuvers respectively for at least 0.4 seconds. Airway resistive pressure (P*resist*) was calculated as the difference between P*peak* and P*plat*. Airway resistance was calculated as P*peak* minus P*plat* divided by inspiratory flow rate. Driving pressure was calculated as P*plat* minus PEEP*tot*. AutoPEEP was calculated as the difference between PEEP*tot and* set PEEP. Static respiratory system compliance (C*stat*) was calculated as V*t* divided by driving pressure.

### Statistical Analysis

Quantitative continuous variables were reported as mean ± standard deviation. Categorical variables were reported as counts and percentages. Descriptive statistics were used to summarize clinical data.

## Results

### Patients, Clinical Characteristics, and Ventilator Settings

Between April 19, 2020 and May 1, 2020, 21 patients were admitted to the medical ICU with ARDS due to COVID-19, 12 of whom were passively ventilated. Patient characteristics are reported in Table 1. The mean age was 58 and 50% (6/12) were men. Most of the subjects were non-smokers (8/12) and had no known history of pulmonary disease (10/12). The average duration between intubation and time of data collection was 1.7 days. Half (6/12) of the patients were receiving NMB at the time of data collection. All were ventilated in the supine position. None were receiving inhaled pulmonary vasodilators or extracorporeal membrane oxygenation (ECMO). One patient received inhaled bronchodilators in the 24 hours preceding data collection. No patients had significant secretions. No prior PFTs were available for any of the subjects. Ventilator settings are summarized in Table 2.

**Table 1.** Demographic and Clinical Characteristics of the Patients.

Patient no.	Age	Sex	BMI	Smoking history	History of COPD/asthma	Other chronic pulmonary diseases	Number of days since intubation
Patient 1	67	F	33.22	Never smoked	None	None	2
Patient 2	62	F	49.04	Unknown	COPD	None	3
Patient 3	59	M	45.64	Former smoker	None	None	3
Patient 4	64	M	32.45	Never smoked	None	None	1
Patient 5	61	M	32.03	Never smoked	None	None	2
Patient 6	61	F	28.12	Never smoked	Asthma	Ehlers-Danlos syndrome	2
Patient 7	44	M	35.26	Unknown	None	None	12
Patient 8	35	M	46.23	Never smoked	None	None	3
Patient 9	74	F	23.56	Never smoked	None	History of pulmonary embolism	0
Patient 10	78	M	27.14	Never smoked	None	None	0
Patient 11	52	F	41.14	Former smoker	None	None	2
Patient 12	43	F	25.09	Never smoked	None	None	1
**Mean (±SD)**	**58 (±13)**		**35 (±9)**				**3 (±3)**

Abbreviations: BMI, body mass index; COPD, chronic obstructive pulmonary disease.

**Table 2.** Ventilator Settings Selected by the Attending Physician or Respiratory Therapist.

Patient no.	Set RR (per minute)	Set V*t* (ml)	Set F*i*O2 (%)	Set PEEP (cm H_2_O)	Set flow rate (L/min)	Minute ventilation (L/min)
Patient 1	34	300	35	14	45	10
Patient 2	30	380	40	12	57	11
Patient 3	32	370	40	12	55	12
Patient 4	32	410	40	8	54	13
Patient 5	32	380	45	12	57	12
Patient 6	30	350	60	14	60	11
Patient 7	36	280	100	15	56	10
Patient 8	24	420	100	15	56	10
Patient 9	32	350	50	10	53	11
Patient 10	32	350	100	12	60	11
Patient 11	30	300	40	14	60	9
Patient 12	32	320	40	14	55	10
**Mean (±SD)**	**31 (±3)**	**351 (±44)**	**58 (±26)**	**13 (±2)**	**56 (±4)**	**11 (±1)**

Abbreviations: F*i*O2, fraction of inspired oxygen; PEEP, positive end expiratory pressure; RR, respiratory rate; V*t*, tidal volume.

### Patient-Ventilator Interaction and Ventilator Waveforms

**Figure 1.**
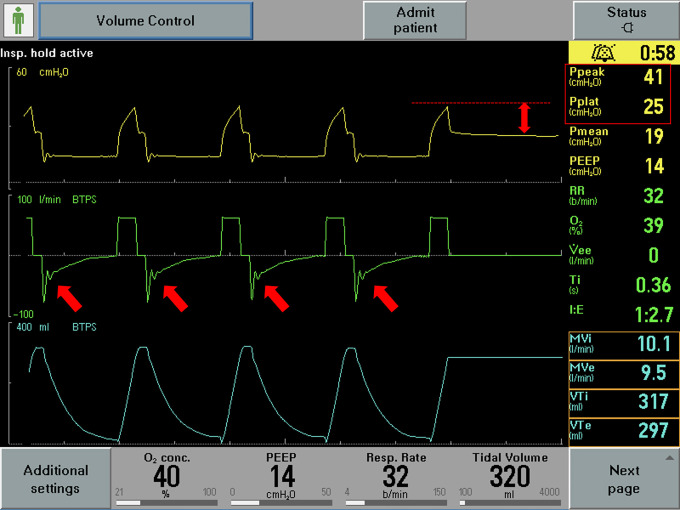
Representative image of ventilator waveforms. The expiratory flow waveform demonstrates rapid flow deceleration followed by slow return to baseline (arrows). Also note the abnormal P*peak* minus P*plat* (double-headed arrow and right-hand side of the pane).

**Figure 2. fig2-08850666211000601:**
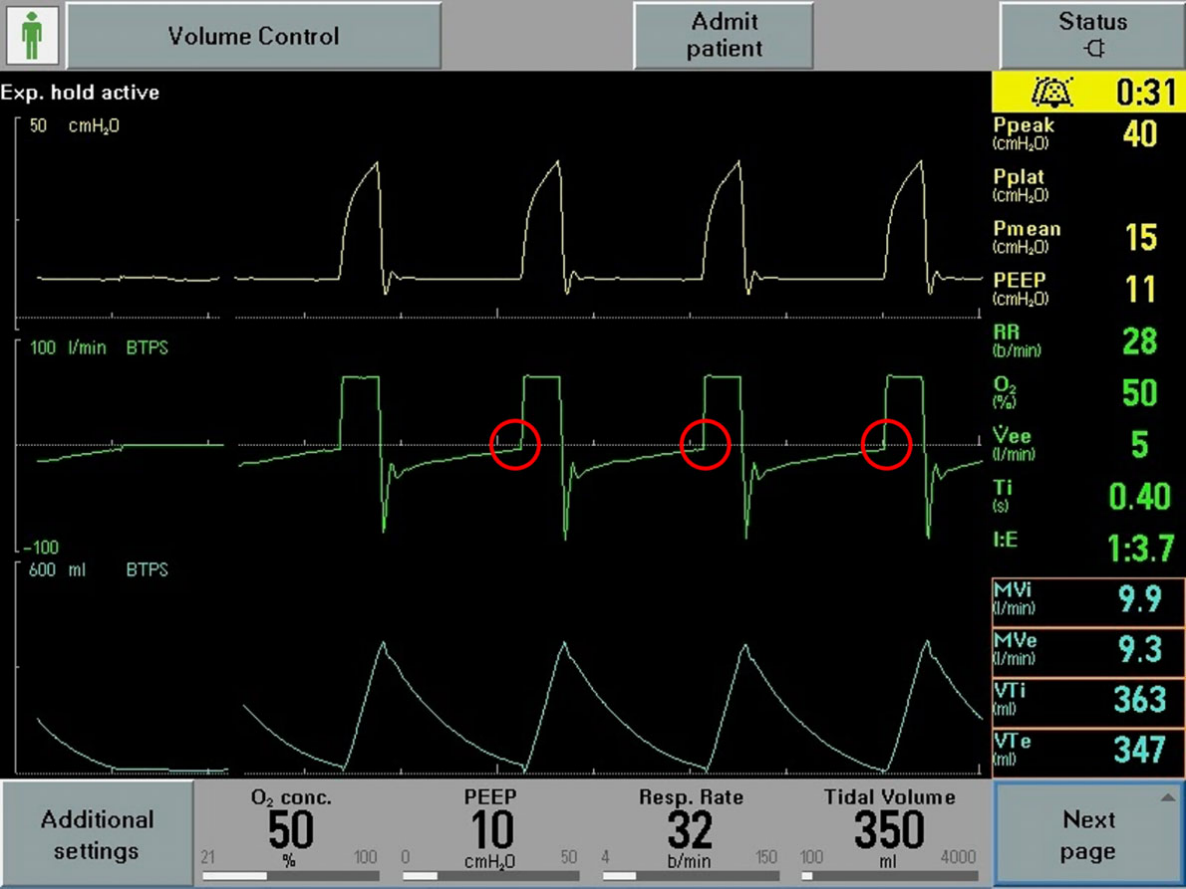
Representative image of ventilator waveforms. Expiratory flow present at end-expiration (circles).

Respiratory system mechanics are shown in Table 3. All patients had increased airway resistance, illustrated by P*peak* and P*resist* that were significantly elevated at 42.3 [±5.26] cm H_2_O and 18.8 [±3.60] cm H_2_O respectively. Calculated airway resistance was markedly increased at 20.4 [±4.13] cm H_2_O/L/sec. AutoPEEP was minimally elevated at 1 [±1] cm H2O. Driving pressure was 9.92 [±2.75] cm H_2_O. Static compliance of the respiratory system was 39.0 [±15.6] ml/cm H_2_O. Examination of the ventilator waveforms, specifically expiratory flow curve, indicated the presence of airflow obstruction (Figures 1 [Fig fig2-08850666211000601]-3).

**Table 3.** Ventilator-Derived Indices of Airway Resistance and Respiratory System Compliance.

Patient no.	P*peak* (cm H2O)	P*plat* (cm H2O)	AutoPEEP (cm H2O)*	P*resist* (cm H2O)*	Airway resistance (cmH2O/L/sec)*	Driving pressure (cm H2O)*	C*stat* (ml/cmH2O)*
Patient 1	45	25	1	20	26.4	10	30
Patient 2	44	23	1	21	22.2	10	38
Patient 3	37	24	0	13	14.4	12	30.83
Patient 4	34	19	4	15	16.8	7	58.57
Patient 5	38	21	4	17	18	5	76
Patient 6	43	27	1	16	16.2	12	29.16
Patient 7	51	29	–1	22	23.4	15	18.67
Patient 8	48	24	0	24	25.71	9	46.67
Patient 9	40	20	1	20	22.86	9	38.89
Patient 10	49	25	1	24	24	12	29.17
Patient 11	38	21	0	17	17	7	42.86
Patient 12	41	25	0	16	17.49	11	29
**Mean (±SD)**	**42 (±5)**	**24 (±3)**	**1 (±1)**	**19 (±4)**	**20 (±4)**	**10 (±3)**	**39 (±16)**

Abbreviations: C*stat*, static compliance; P*peak*, peak pressure; P*plat*, plateau pressure; P*resist*, resistive pressure.

* Calculation of indices: AutoPEEP = intrinsic PEEP – extrinsic PEEP; P*resist* = P*peak* – P*plat*; airway resistance = (P*peak* – P*resist*)/flow rate; driving pressure = P*plat* – intrinsic PEEP; C*stat* = V*t*/driving pressure.

**Figure 3.**
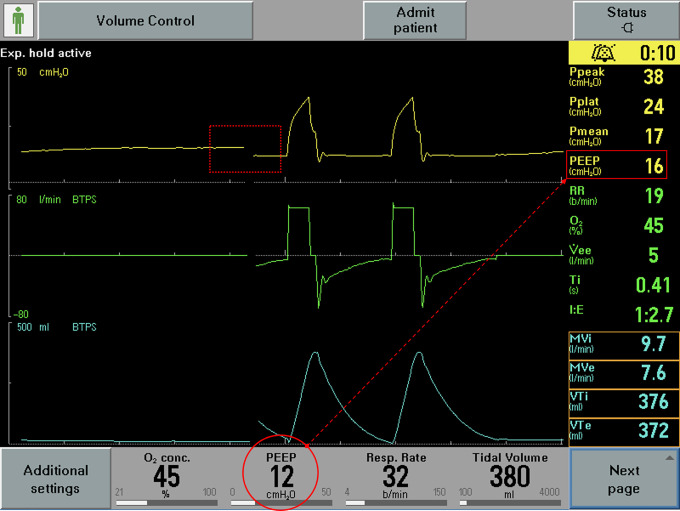
Representative image of ventilator waveforms. Intrinsic PEEP unmasked by manual expiratory hold maneuver (dotted rectangle; circle at bottom of the pane represents operator set PEEP and rectangle on the right-hand side of the pane represents PEEP*tot*).

## Discussion

In mechanically ventilated patients, breath-by-breath measurement of pressure and flow allows characterization of the mechanical properties of the respiratory system, such as compliance and resistance. Such measurements revealed an unusual finding in our patients with COVID-19 ARDS: airways resistance was elevated in all subjects. This novel finding has not been previously reported to our knowledge. Airways resistance is modestly elevated in ARDS,^9^ but generally not to the degree seen in our patients.

Our findings show that peak pressures were significantly elevated in all subjects. Further, this was from a combination of both decreased compliance leading to elevated plateau pressures, and increased resistance to airflow. The latter is indicated by the elevated P*resist* and airway resistance. While decreased compliance would be an expected finding in ARDS secondary to COVID-19, the elevated airway resistance to this degree is unusual and unexpected. Although there was individual variation in the magnitude of resistive indices, they were elevated in all subjects.

There are several challenges which make interpretation of the results difficult—most importantly, lack of a control group. Additionally, we considered the possibility that our findings were due to the effects of the ventilator circuit. We assessed whether the HME filter, used to filter viral particles, was responsible for the abnormal resistance. Older literature reported increased airway resistance with the use of filters in ventilator circuits, especially with high minute ventilation.^10,11^ The manufacturer of the filter we use (Intersurgical ltd, Inter-Therm^TM^ range, model number: 1341031 S)^12^ claims that it adds a resistance of 2.7 cmH_2_O at a flow rate of 60 L/min. The mean flow rate for our subjects was 56 L/min, therefore only a small fraction of the resistive pressure we measured can be accounted for by the filter. Moreover, in one non-study subject, we briefly removed the HME filter to determine its impact, finding little change in P*peak*, P*resist*, or expiratory flow shape. We also conducted an experiment using the HME filter on a simulator (Michigan Instruments 5600i Dual Adult TTL Training/Test Lung) at 2 different flow rates and found that the filter added a resistance of 2 cmH_2_O/L/sec at any given flow. The endotracheal tube itself presents a resistive load, but this would be insufficient to explain resistance of sufficient magnitude.^13^ In our cohort of patients, the smallest size of endotracheal tube used was 7 millimeters (internal diameter). We assume that increased P*resist* reflects an increase in the Ohmic component of resistance rather than the viscoelastic fraction. We believe it unlikely that substantially increased viscoelastic resistance accounts for our findings since we used a rather brief (0.4 s) end-inspiratory pause. Given the complexity of patient-ventilator interactions, further confirmatory studies are needed to address the issue of airway resistance in patients ventilated with heated humidification systems.

An alternative explanation to account for our finding of slowed expiratory flow is expiratory braking. The post-inspiratory complex, which controls the transition from inspiration to expiration, could act to limit expiratory flow, mimicking expiratory airflow obstruction.^14,15^ SARS-CoV-2 is a neurotropic virus and could impact the respiratory centers directly. We think this explanation is unlikely since it would not also explain the elevation in inspiratory P*resist*.

Finally, the mean BMI of patients in our study is 35. Airways resistance is modestly elevated in obesity,^16^ but this cannot explain the degree of elevation in our patients, nor account for the presence of obstruction in all of them.

We believe it is more likely that increased airways resistance is an intrinsic feature of severe COVID-19 lung disease. There is precedent for viral pneumonias to produce a resistive lesion, most notably with infection due to respiratory syncytial virus.^17^ The anatomic site of obstruction is uncertain, although some patients have increased bronchomotor tone. Others exhibit airflow obstruction due to neutrophil extracellular traps (NETs), webs of extracellular chromatin, microbicidal proteins, and oxidant enzymes in small airways, as has been seen in RSV and other pneumonias.^18,19^ Evidence for the presence of NETs in patients with COVID-19, especially in those with more severe disease, has been published. Further, serum from patients with COVID-19 triggers NET release from control neutrophils in vitro.^20^

Radiographic surveys of patients with COVID-19 have identified airway abnormalities, including bronchial wall thickening.^21^ These findings are more evident in the most severely afflicted patients.^22^ Few autopsy studies have been published, but peribronchial lymphocytic accumulation, connective tissue within bronchioles, and granulocytic infiltration of bronchi have been described (in addition to other findings).^23,24^ Each of these provides a potential structural basis for the physiological findings we describe.

Recognition of airflow obstruction in COVID-19 pneumonia has potential implications for therapy. Heliox lowers the resistive pressure where flow is turbulent,^25–27^ and can ameliorate respiratory distress in infants with RSV bronchiolitis.^28^ One case report describes successful treatment of airflow obstruction associated with coronavirus OC43 in a child with high-flow heliox administration.^29^ Heliox is most effective when the fraction of helium is high, but the modest degree of hypoxemia seen in many COVID-19 patients may allow its use. If NETs play a causal role in the pathogenesis of COVID-19 respiratory failure, aerosolized dornase might bear consideration,^30^ as has been reported in other subtypes of ARDS.^31^ Bronchodilator use, preferably via in-line metered dose inhalers, could also be considered, especially when significant airflow obstruction interferes with ventilation (for example, when there is evidence of developing auto-PEEP). Recently, a case series showed excellent tolerability of nebulized in-line dornase and albuterol in mechanically ventilated patients with COVID-19.^32^

Not long after the initial description of ARDS, John Murray warned against lumping multiple diseases into one syndrome,^33^ thereby “detracting from important and distinctive differences in pathogenesis, therapy, and prognosis.” Forty-five years later, and only months following Dr. Murray’s death from COVID-19, his words ring true.

## Conclusion

Severe respiratory failure due to COVID-19 is regularly associated with airflow obstruction. This may have implications for the pathogenesis of respiratory failure and suggests potential therapies.

## References

[1] Zhu N Zhang D Wang W, et al. A novel coronavirus from patients with pneumonia in China, 2019. N Engl J Med. 2020;382(8):727–733. 31978945 10.1056/NEJMoa2001017 PMC7092803

[2] Guan WJ Ni ZY Hu Y, et al. Clinical characteristics of Coronavirus disease 2019 in China. N Engl J Med. 2020;382(18):1708–1720. 32109013 10.1056/NEJMoa2002032 PMC7092819

[3] Bhatraju PK Ghassemieh BJ Nichols M, et al. Covid-19 in critically ill patients in the Seattle region—case series. N Engl J Med. 2020;382(21):2012–2022. 32227758 10.1056/NEJMoa2004500 PMC7143164

[4] Grasselli G Zangrillo A Zanella A, et al. Baseline characteristics and outcomes of 1591 patients infected with SARS-CoV-2 admitted to ICUs of the Lombardy region, Italy. JAMA. 2020;323(16):1574–1581. 32250385 10.1001/jama.2020.5394 PMC7136855

[5] Force ADT Ranieri VM Rubenfeld GD, et al. Acute respiratory distress syndrome: the Berlin definition. JAMA. 2012;307(23):2526–2533. 22797452 10.1001/jama.2012.5669

[6] Tobin MJ. Basing respiratory management of coronavirus on physiological principles. Am J Respir Crit Care Med. 2020;201(11):1319–1320. 32281885 10.1164/rccm.202004-1076ED PMC7258630

[7] Gattinoni L Coppola S Cressoni M Busana M Rossi S Chiumello D. COVID-19 does not lead to a “typical” acute respiratory distress syndrome. Am J Respir Crit Care Med. 2020;201(10):1299–1300. 32228035 10.1164/rccm.202003-0817LE PMC7233352

[8] Gattinoni L Chiumello D Caironi P, et al. COVID-19 pneumonia: different respiratory treatments for different phenotypes? Intensive Care Med. 2020;46(6):1099–1102. 32291463 10.1007/s00134-020-06033-2 PMC7154064

[9] Wright PE Bernard GR. The role of airflow resistance in patients with the adult respiratory distress syndrome. Am Rev Respir Dis. 1989;139(5):1169–1174. 2653150 10.1164/ajrccm/139.5.1169

[10] Buckley PM. Increase in resistance of in-line breathing filters in humidified air. Br J Anaesth. 1984;56(6):637–643. 6586196 10.1093/bja/56.6.637

[11] Cohen IL Weinberg PF Fein IA Rowinski GS. Endotracheal tube occlusion associated with the use of heat and moisture exchangers in the intensive care unit. Crit Care Med. 1988;16(3):277–279. 3422625 10.1097/00003246-198803000-00013

[12] Intersurgical. Inter-Therm™ and Inter-Therm™ Mini. Published August 2019. Accessed February 26, 2021. https://us.intersurgical.com/content/files/113418/658477610.

[13] Wright PE Marini JJ Bernard GR. In vitro versus in vivo comparison of endotracheal tube airflow resistance. Am Rev Respir Dis. 1989;140(1):10–16. 2751156 10.1164/ajrccm/140.1.10

[14] Jonkman AH de Vries HJ Heunks LMA. Physiology of the respiratory drive in ICU patients: implications for diagnosis and treatment. Crit Care. 2020;24(1):104. 32204710 10.1186/s13054-020-2776-z PMC7092542

[15] Pellegrini M Hedenstierna G Roneus A Segelsjö M Larsson A Perchiazzi G. The diaphragm acts as a brake during expiration to prevent lung collapse. Am J Respir Crit Care Med. 2017;195(12):1608–1616. 27922742 10.1164/rccm.201605-0992OC

[16] Pelosi P Croci M Ravagnan I, et al. The effects of body mass on lung volumes, respiratory mechanics, and gas exchange during general anesthesia. Anesth Analg. 1998;87(3):654–660. 9728848 10.1097/00000539-199809000-00031

[17] Hall WJ Hall CB Speers DM. Respiratory syncytial virus infection in adults: clinical, virologic, and serial pulmonary function studies. Ann Intern Med. 1978;88(2):203–205. 415653 10.7326/0003-4819-88-2-203

[18] Cortjens B de Boer OJ de Jong R, et al. Neutrophil extracellular traps cause airway obstruction during respiratory syncytial virus disease. J Pathol. 2016;238(3):401–411. 26468056 10.1002/path.4660

[19] Cortjens B de Jong R Bonsing JG van Woensel JBM Antonis AFG Bem RA. Local dornase alfa treatment reduces NETs-induced airway obstruction during severe RSV infection. Thorax. 2018;73(6):578–580. 28780505 10.1136/thoraxjnl-2017-210289

[20] Zuo Y Yalavarthi S Shi H, et al. Neutrophil extracellular traps in COVID-19. JCI Insight. 2020;5(11):e138999. 10.1172/jci.insight.138999 PMC7308057 32329756

[21] Ye Z Zhang Y Wang Y Huang Z Song B. Chest CT manifestations of new coronavirus disease 2019 (COVID-19): a pictorial review. Eur Radiol. 2020;30(8):4381–4389. 32193638 10.1007/s00330-020-06801-0 PMC7088323

[22] Li K Wu J Wu F, et al. The clinical and chest CT features associated with severe and critical COVID-19 pneumonia. Invest Radiol. 2020;55(6):327–331. 32118615 10.1097/RLI.0000000000000672 PMC7147273

[23] Copin MC Parmentier E Duburcq T Poissy J Mathieu D. Time to consider histologic pattern of lung injury to treat critically ill patients with COVID-19 infection. Intensive Care Med. 2020;46(6):1124–1126. 32328726 10.1007/s00134-020-06057-8 PMC7178098

[24] Wichmann D Sperhake JP Lütgehetmann M, et al. Autopsy findings and venous thromboembolism in patients With COVID-19. Ann Intern Med. 2020;173(12):1030. 33316197 10.7326/L20-1206

[25] Kudukis TM Manthous CA Schmidt GA Hall JB Wylam ME. Inhaled helium-oxygen revisited: effect of inhaled helium-oxygen during the treatment of status asthmaticus in children. J Pediatr. 1997;130(2):217–224. 9042123 10.1016/s0022-3476(97)70346-9

[26] Manthous CA Hall JB Caputo MA, et al. Heliox improves pulsus paradoxus and peak expiratory flow in nonintubated patients with severe asthma. Am J Respir Crit Care Med. 1995;151(2 Pt 1):310–314. 7842183 10.1164/ajrccm.151.2.7842183

[27] Mink SN Wood LD. How does HeO2 increase maximum expiratory flow in human lungs? J Clin Invest. 1980;66(4):720–729. 7419718 10.1172/JCI109909 PMC371646

[28] Liet JM Ducruet T Gupta V Cambonie G. Heliox inhalation therapy for bronchiolitis in infants. Cochrane Database Syst Rev. 2015;(9):Cd006915. 26384333 10.1002/14651858.CD006915.pub3 PMC8504435

[29] Morgan SE Vukin K Mosakowski S, et al. Use of heliox delivered via high-flow nasal cannula to treat an infant with coronavirus-related respiratory infection and severe acute air-flow obstruction. Respir Care. 2014;59(11):e166–170. 25118308 10.4187/respcare.02728

[30] Earhart AP Holliday ZM Hofmann HV Schrum AG. Consideration of dornase alfa for the treatment of severe COVID-19 acute respiratory distress syndrome. New Microbes New Infect. 2020;35:100689. 32355564 10.1016/j.nmni.2020.100689 PMC7192073

[31] Pottecher J Noll E Borel M, et al. Protocol for TRAUMADORNASE: a prospective, randomized, multicentre, double-blinded, placebo-controlled clinical trial of aerosolized dornase alfa to reduce the incidence of moderate-to-severe hypoxaemia in ventilated trauma patients. Trials. 2020;21(1):274. 32183886 10.1186/s13063-020-4141-6 PMC7079402

[32] Weber AG Chau AS Egeblad M, et al. Nebulized in-line endotracheal dornase alfa and albuterol administered to mechanically ventilated COVID-19 patients: a case series. Mol Med. 2020;26(1):91. 32993479 10.1186/s10020-020-00215-w PMC7522910

[33] Murray JF. Editorial: the adult respiratory distress syndrome (may it rest in peace). Am Rev Respir Dis. 1975;111(6):716–718. 1173542 10.1164/arrd.1975.111.6.716

